# A Workflow for Improved Analysis of Cross-Linking Mass Spectrometry Data Integrating Parallel Accumulation-Serial Fragmentation with MeroX and Skyline

**DOI:** 10.1021/acs.analchem.4c00829

**Published:** 2024-05-02

**Authors:** Juan Camilo Rojas Echeverri, Frank Hause, Claudio Iacobucci, Christian H. Ihling, Dirk Tänzler, Nicholas Shulman, Michael Riffle, Brendan X. MacLean, Andrea Sinz

**Affiliations:** † Department of Pharmaceutical Chemistry and Bioanalytics, Martin-Luther-University Halle-Wittenberg, 06120 Halle, Germany; ‡ Center for Structural Mass Spectrometry, Martin-Luther-University Halle-Wittenberg, 06120 Halle, Germany; § Institute for Molecular Medicine, Martin-Luther-University Halle-Wittenberg, 06120 Halle, Germany; ∥ Department of Physical and Chemical Sciences, University of L’Aquila, 67100 L’Aquila, Italy; ⊥ Department of Genome Sciences, University of Washington, Seattle, Washington 98195, United States; # Department of Biochemistry, University of Washington, Seattle, Washington 98195, United States

## Abstract

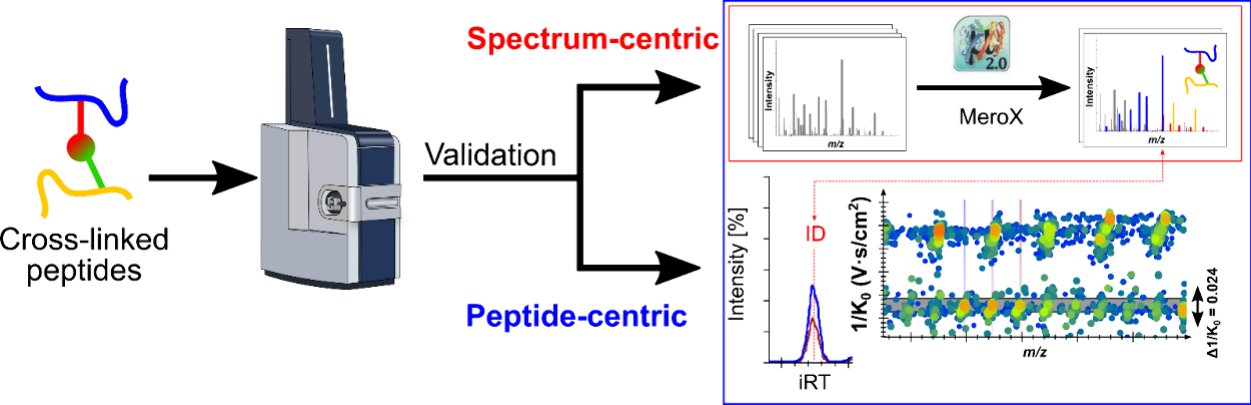

Cross-linking mass spectrometry (XL-MS) has evolved into a pivotal technique for probing protein interactions. This study describes the implementation of Parallel Accumulation-Serial Fragmentation (PASEF) on timsTOF instruments, enhancing the detection and analysis of protein interactions by XL-MS. Addressing the challenges in XL-MS, such as the interpretation of complex spectra, low abundant cross-linked peptides, and a data acquisition bias, our current study integrates a peptide-centric approach for the analysis of XL-MS data and presents the foundation for integrating data-independent acquisition (DIA) in XL-MS with a vendor-neutral and open-source platform. A novel workflow is described for processing data-dependent acquisition (DDA) of PASEF-derived information. For this, software by Bruker Daltonics is used, enabling the conversion of these data into a format that is compatible with MeroX and Skyline software tools. Our approach significantly improves the identification of cross-linked products from complex mixtures, allowing the XL-MS community to overcome current analytical limitations.

## Introduction

Recent advancements in mass spectrometry (MS) have transformed our understanding of protein function, particularly through techniques like cross-linking mass spectrometry (XL-MS).^1−3^ XL-MS provides unique insights into protein conformations and protein–protein interactions.^4,5^ Despite its promise, several technical and analytical challenges persist, hindering its full utilization in protein interaction studies: (I) Cross-linked (XL) products are present at significantly lower concentrations than non-XL species, demanding sensitive MS techniques with high dynamic range for detection and quantification. This requires specific enrichment strategies due to the background noise from more abundant species.^1,2,5^ (II) Fragment ion spectra from XL products often exhibit overlapping series and diagnostic ions from cross-linkers, complicating their unambiguous identification. Specialized analytical strategies and computational tools are needed for accurate interpretation. (III) The ambiguity of the XL site adds an additional layer of complexity, since the presence of multiple potential XL sites introduces uncertainties in determining the precise interaction interfaces.^6^ (IV) Data-dependent acquisition (DDA) methods, commonly used in XL-MS, may overlook XL products present in the low-intensity range due to their bias towards more abundant species. This could result in underrepresentation of important protein interaction events.^7^

To address these challenges, targeted and sensitive data acquisition methods like parallel reaction monitoring (PRM) have been adopted. PRM tracks all fragment ions from a limited list of known XL products, improving detection accuracy.^8^ Additionally, data-independent acquisition (DIA) techniques have emerged as an alternative solution. DIA enables systematic and unbiased sampling across the mass range, enhancing sensitivity and reproducibility, crucial for detecting low-abundance species and transient interactions. DIA generates chimeric spectra complicating the assignment of fragment ions especially in complex XL product ion spectra, which can be considered chimeric themselves. Although DDA spectral libraries remain necessary for accurate XL identification, recent studies have shown that with the support of DDA spectral libraries, DIA can enhance the sensitivity and reproducibility of XL detection.^9,10^

The implementation of Parallel Accumulation-Serial Fragmentation (PASEF)^11^ on timsTOF instruments (Bruker Daltonics) has significantly improved ion sampling efficiency, leading to successful applications of PASEF in XL-MS analysis.^12,13^ However, the inherent limitations of biased sampling in DDA persist, necessitating critical validation of raw MS data with visualization tools like Skyline.^14^ Despite these advancements, the integration of techniques, such as trapped ion mobility spectrometry (TIMS), with DDA-PASEF introduces additional complexity by adding an additional dimension of separation where at each point in time a two-dimensional (*m*/*z* vs intensity) data representation becomes three-dimensional (*m*/*z* vs intensity vs ion mobility). This complexity often results in limited software support for native DDA-PASEF data, hindering widespread adoption of these techniques in XL-MS studies.

This technical note details a workflow to process DDA-PASEF data using the native Bruker software infrastructure. First, the LC-TIMS-MS/MS data was processed and simplified into peak lists in mascot generic format (.MGF), compatible with most database search engines. These files are then used for XL product identification by MeroX^15^ and combined with standardized ProXL XML^16^ structure to generate spectral libraries for subsequent analysis of raw MS data with Skyline. This case study, involving bovine serum albumin (BSA) cross-linked with disuccinimidyl dibutyric urea (DSBU) addresses specifics for XL-MS studies using timsTOF instruments. The outlined methodologies aim to empower the cross-linking community with tools to overcome current limitations in DDA applications and establish a framework for quantitative analysis of PRM and DIA XL-MS data with Skyline’s native infrastructure. Additionally, by sharing results in Panorama Public^17^ we aim to promote transparency and standardization in data sharing, crucial for enhancing detection, analysis, and understanding of protein interactions.^18^

## Materials and Methods

### Cross-Linking and Digestion

BSA cross-linking with DSBU was carried out as previously described.^12^ A 10 μM BSA solution in 50 mM 2-[4-(2-hydroxyethyl)-piperazine-1-yl]ethanesulfonic acid (HEPES) (pH 7.5) underwent cross-linking at room temperature for 1 h using a 50-fold molar excess of DSBU, freshly dissolved in DMSO at 25 mM. Alongside, a negative control without DSBU was prepared. Post cross-linking, three 80 μg aliquots from each sample were dried using a SpeedVac concentrator. Sample preparation, including protein digestion, was performed using Suspension-Trapping (S-Trap, Protifi) according to the manufacturer’s guidelines.

### LC-TIMS-MS/MS Data Collection

Dried peptides were reconstituted in 320 μL of 30% (v/v) acetonitrile (ACN) with 0.1% (v/v) trifluoroacetic acid (TFA), adjusting to a concentration of 0.25 μg/μL. From this, a 20 μL aliquot (5 μg) was mixed with 250 fmol of Pierce indexed retention time (iRT) standards and further diluted to 200 μL with 0.1% TFA. For analysis, 40 μL (1 μg of BSA-DSBU digest and 50 fmol of iRT peptides) were loaded onto an UltiMate 3000 RSLC nano-HPLC system (Thermo Fisher Scientific), coupled to a timsTOF Pro mass spectrometer (Bruker Daltonics). Peptides were trapped on a C18 precolumn (precolumn Acclaim PepMap 100, 300 μm × 5 mm, 5 μm, 100 Å, Thermo Fisher Scientific) and separated on a self-packed Picofrit (New Objective) nanospray emitter (360 μm OD × 75 μm ID x 150 mm L, 15 μm Tip ID) with C18-stationary phase (3.0 μm, 120 Å, Dr. Maisch GmbH). The precolumn was washed for 15 min with 0.1% (v/v) TFA at 30 μL/min and 50 °C. Elution and separation were conducted at a flow rate of 300 nL/min using a 90 min linear gradient of water–ACN (3% to 50% B), where A is 0.1% (v/v) formic acid and B is 0.1% (v/v) formic acid in ACN.

After chromatographic separation, peptides were ionized using electrospray ionization (ESI) at a capillary voltage of 1500 V, with drying facilitated by N_2_ gas at 180 °C and a flow rate of 3.0 L/min. The ions were then analyzed using trapped ion mobility spectrometry (TIMS) in a dual cell setup before tandem mass spectrometry (MS/MS) detection. TIMS-MS/MS data acquisition utilized DDA-PASEF with ion accumulation and ramp time set to 200 ms. Three mobility-dependent collision energy ramps were employed (see Table 1).

**Table 1:** Ion Mobility Dependent Collision Energy Profiles

**Settings**	**CE Start (eV)**	**CE End (eV)**	**1/K**_**0**_**Start(Vs/cm**^**2**^**)**	**1/K**_**0**_**End(Vs/cm**^**2**^**)**
**Low CE Profile**	59	23	1.60	0.73
**Mid CE Profile**	75	23	1.60	0.73
**High CE Profile**	95	23	1.60	0.73

Collision energies were linearly interpolated between specified 1/K_0_ values, remaining constant above or below these values. The PASEF precursor target intensity was set to 100,000, with a minimum intensity threshold of 1,000. Each acquisition cycle, lasting 2.47 s, triggered 10 PASEF MS/MS scans. Precursor ions with *m*/*z* ranging from 100 to 1700 and charge states from 3+ to 8+ were chosen for fragmentation. Active exclusion, set for 0.5 min with a mass width of 0.015 Th and 1/K_0_ width of 0.100 V•s•cm^–2^, included early retargeting if precursor intensity improved by 4x.

### Data Conversion, XL Peptide Identification, and Data Validation

Post acquisition, DDA-PASEF data was processed using DataAnalysis (v5.3; Bruker Daltonics) to create peak lists of fragment ion spectra in MGF files. Fragment ion spectra with precursor ions collected within a 0.75 min window, having a monoisotopic *m*/*z* deviation within 0.015, and 1/K_0_ values within 0.025 V•s•cm^–2^, were combined.

Identification of cross-links was conducted using MeroX (v. 2.0.1.7)^15^ and SwissProt BSA sequence (Uniprot ID: P02769). BSA-DSBU XL annotation settings included: fully specific proteolytic cleavage at Lys and Arg (up to 3 missed cleavages, peptide lengths 5–50 amino acids), post-translational modifications (PTMs) were set as alkylation of Cys by iodoacetamide (fixed) and oxidation of Met (variable), cross-linker specificity (Lys, Ser, Thr, Tyr, N-terminus), XL-fragments (essential: Bu + C_4_H_7_NO, BuUr + C_5_H_5_NO_2_; optional: Δmass = 0), search algorithm: RISEUP mode: up to two missing ions, a-, b-, y-ion series, precursor mass accuracy (10 ppm), fragment ion mass accuracy (15 ppm), 10% intensity prescore cutoff, 25% false discovery rate (FDR) cutoff, and minimum score cutoff of 15. After searches were done, files were combined and the global FDR was set to 5%.

MeroX results from each analysis were merged into a single data set and exported as CSV files. An in-house R script was used to extract a peptide and precursor-specific ion mobility library from these grouped results (see R-notebook deposited in https://panoramaweb.org/XL-MS_MeroX_Skyline.url). For each matched precursor ion, mean 1/K_0_ and range were calculated based on all reported 1/K_0_ values from the XL spectra matches (XSMs). These data were formatted into a Skyline ion mobility spectral library and exported as CSV files.

### Peptide ID Import and Validation in Skyline

MeroX results, converted to ProXL XML files (https://github.com/yeastrc/proxl-import-merox), along with MGF files, were used to create spectral libraries of XL products in Skyline. Pierce iRT standards, measured separately, provided retention time calibration for the BSA-DSBU + Pierce iRT peptide analysis. The compiled ion mobility library was integrated into the document to generate extracted ion chromatograms (EICs) of the first three isotopes of each precursor ion from raw DDA-PASEF files, using 10 ppm extraction windows. XLs meeting criteria−5% FDR at the XSM level, detection across all sample replicates, and absence in negative controls−were confirmed as true. Their retention times, adjusted using Pierce iRT standards in Skyline’s retention time calculator, were then indexed. Raw data are publicly available with ProteomeXchange identifier PXD047307 and Panorama Public under https://panoramaweb.org/XL-MS_MeroX_Skyline.url.

## Results and Discussion

### Data Analysis Workflow

**Figure 1:**
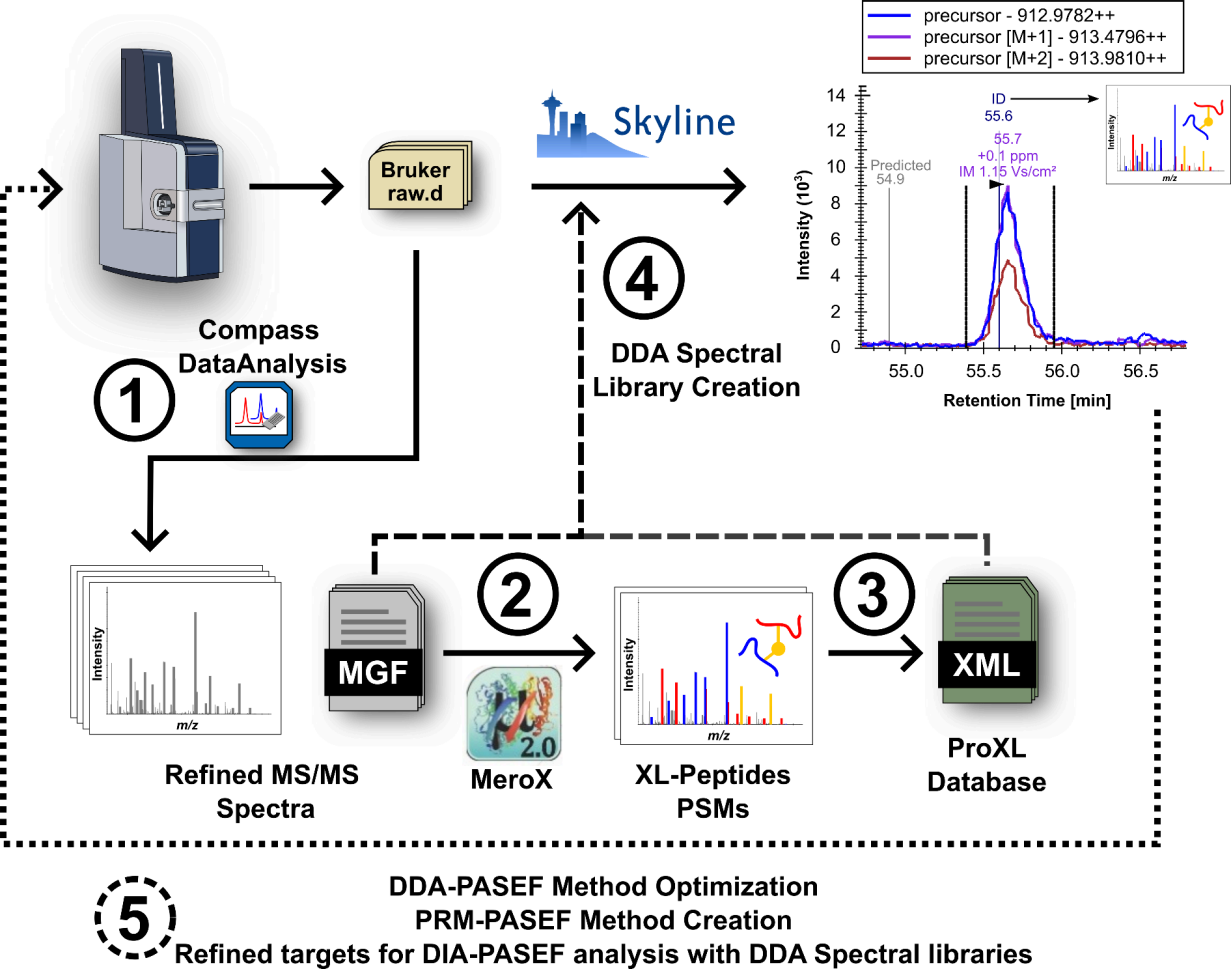
Data analysis workflow. Raw LC-TIMS-MS/MS data was preprocessed with DataAnalysis to create peak lists in MGF format. The MGF files were used as input for XL peptides identification with MeroX 2.0. MeroX results were converted into ProXL format. The ProXL files and MGF files were used as input for creating spectral libraries with Skyline.

This workflow utilizes DataAnalysis (Bruker Daltonics) tools to process native LC-TIMS-MS/MS data produced by timsTOF instruments (Figure 1). It begins with mass recalibration and compound detection, associating each compound with fragment ion spectra for a specific *m*/*z* within a certain retention time range and *m*/*z* error tolerance. Mass spectra are summed and peak picked to derive average precursor and fragment ion spectra. Additionally, for PASEF data, the average precursor scan is used to determine the ion’s charge state, monoisotopic peak *m*/*z*, and the ion mobility center (1/K_0_). The latter is done by creating an ion mobilogram across the same retention time range. Fragment ion TOF scans across PASEF ramps, falling within the selected *m*/*z*, retention time, and 1/K_0_ ranges are associated accordingly. The resulting summed and peak picked fragment ion spectra are exported as a simplified MGF file where the precursor ion’s 1/K_0_ is annotated in the scan title. By leveraging Bruker software’s native capabilities, this approach efficiently processes TIMS-MS/MS data, exploiting improvements in sensitivity and selectivity using PASEF, and circumvents limitations in other software tools like MeroX that cannot process raw LC-IMS-MS/MS data. Although demonstrated for XL-MS, this portion of the workflow is broadly applicable to proteomics, as most DDA database search engines can utilize these MGF files.

Skyline’s support for interpreting XLs has significantly advanced since previous XL-MS implementations.^7,8^ Extracting peptide ion mobility libraries from MeroX results now enables streamlined analysis of native DDA-PASEF and DIA-PASEF data in Skyline. Previously, XLs were imported into Skyline as artificial linearized entities with mass shifts from cross-linkers represented by artificial modifications or use of the “molecule” interface of the software. These methods, however, led to ambiguity in XL site reporting and limited use of Skyline’s native peptide fragment ion calculation infrastructure. Recent community discussions have highlighted the need for standardization in XL-MS reporting.^18^ Therefore, Skyline has undergone several improvements to align with these standardization goals. Enhancements include better native support for calculating XL peptide fragment ions, including those from MS-cleavable cross-linkers, and a mandate to report cross-linking results in ProXL format, a standardized XML format that already supports multiple XL-MS database search engines.

MGF files are utilized for XLs identification using MeroX, which are then parsed into ProXL XML files. These ProXL files guide the BiblioSpec function in Skyline to map MGF-stored fragment ion spectra to XL proposals, creating a spectral library. This library, along with indexed retention times (iRTs) facilitates analysis of XL-MS data with targeted and untargeted workflows that have already been developed for other bottom-up proteomics applications. Accompanying this technical note is a detailed tutorial on applying this workflow to a well-characterized XL-MS benchmark system,^19^ BSA (see Supporting Information or https://panoramaweb.org/XL-MS_MeroX_Skyline.url). While compatible with all LC-MS/MS platforms, additional information is provided for processing data from timsTOF instruments.

### Beyond Spectrum-Centric Validation in XL-MS

Traditionally, XL-MS has employed a “spectrum-centric” approach,^20^ where peptide identification relies on a reliable XSM. With this approach only peptide sequences that are derived from a match to a fragment ion spectrum are considered to be present in a particular sample. When replicates are available, it has been shown that IDs shared in multiple replicates tend to be the most reliable and can control FDR efficiently.^21^ However, this raises questions about the reliability of XSMs that, despite passing global FDR filtering, are found only in some of the technical replicates.^15^ When considering the incomplete sampling limitations of DDA, it is incorrect to assume that a peptide is not present in a sample if no XSM exists. The corresponding precursor ions, although present, might simply not have been selected for fragmentation. This consideration is particularly relevant for low abundance signals, such as those of XLs, amidst intense background. Therefore, the XL-MS community has developed further validation approaches that leverage available protein models to use XL distance constraints as false positive discriminators.^22,23^ Although this approach proves its usefulness for large proteome-wide data sets, it is limited by the availability and accuracy of protein models and is ineffective for intrinsically disordered proteins or regions with flexible conformation ensembles that defy standard modeling and distance analysis.^24^

This technical note proposes a complementary validation approach for XLs identified with data acquired in DDA mode, adopting strategies from “peptide-centric” methods^20^ used in DIA and targeted data analysis. Although the list of XLs that are tested for detection still depends on spectrum-centric search engines such as MeroX, here we show how to use the complete LC-(TIMS)-MS/MS data collected to test detection and alignment of identifications proposed from any replicate measurement. This approach assumes each peptide elutes once during its chromatographic separation, collecting precursor and fragment ion spectra from which a diversity of data can be collected such as apex retention time, peak shape, coelution of isotopes, precursor ion’s mass error, isotopic pattern, ion mobility, and XSMs. All of this information is weighed together to make a decision on whether the peptide was detected or not either through automatic chromatographic peak peaking models or manual evaluation. For this purpose, Skyline has been chosen due to its versatility in visualizing LC-TIMS-MS/MS data and promotion of transparency in data analysis by enabling sharing results to public databases like Panorama.^17^

**Figure 2:**
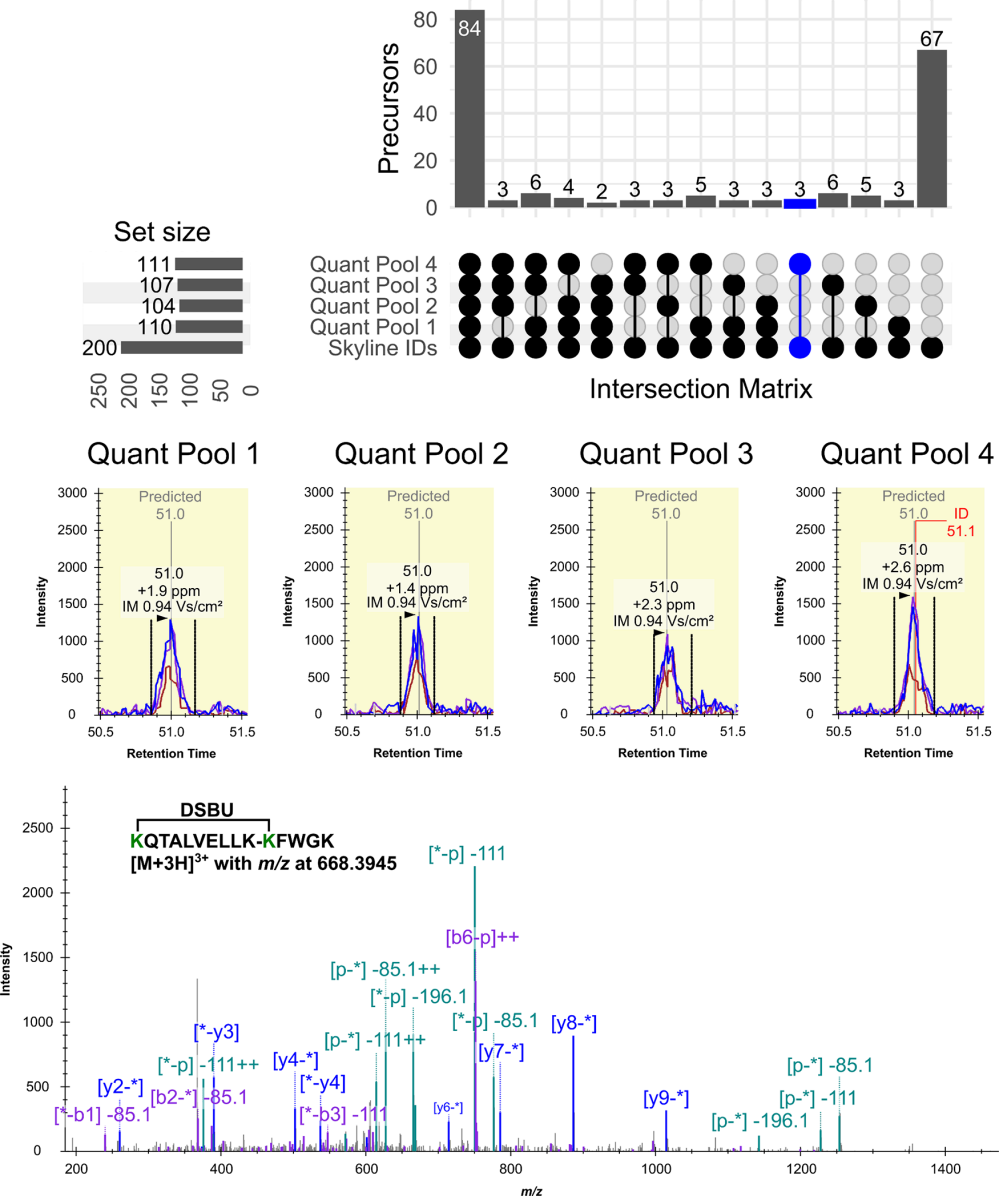
Evaluation of identification reproducibility of precursor ions associated with. UpSet plot representing the overlap of identified precursor ions (top panel) across replicate injections of a pooled digest of cross-linked BSA with DSBU. The identified precursors per replicate are contrasted with a list validated using results from all measurements in this data set. Blue highlight corresponds to the precursor ions carrying XSMs only in “Low CE 4” sample. Extracted ion chromatograms of the [M+3H]^3+^ ion of the KQTALVELLK-KFWGK-(DSBU@1,1) XL (middle panel) which was identified by MeroX only in replicate “Low CE 4” and corresponding XSM (bottom panel).

This paradigm shift is particularly useful in simplified in vitro systems with limited XL diversity for accurate FDR measurement at the peptide and interprotein levels. It also compensates for the stochastic nature of DDA while at the same time allows aligning results acquired with different instrument settings or acquisition modes.^25^ An example is illustrated in Figure 2, where an UpSet plot^26^ is used to contrast IDs from four technical replicates, measured with a low collision energy profile (Low CE), with a list of validated precursor ions of XLs. This analysis reveals that while only 42% of identified precursor ions carried XSMs across all technical replicates (Figure 2; top panel), EICs of peptides with XSMs in a single replicate still showed matching chromatographic peaks, ion mobility, and retention time. This is exemplified with the [M+3H]^3+^ ion of the KQTALVELLK-KFWGK-(DSBU@1,1) XL (Figure 2; middle panel). Notably, the EICs of this ion were distinguishable despite their low signal-to-noise (S/N) ratio. Only, through the summation of multiple TOF scans across the PASEF ramps collected across this chromatographic peak an interpretable fragment ion spectrum was still available for one of the replicates (Figure 2; bottom panel).

Additionally, the UpSet plot reveals that a subset of 67 precursor ions that were not identified in the technical replicates (Low CE) were, however, identified in samples subjected to higher collision energies. These ions required increased collision energies for effective fragmentation, generating interpretable spectra. In contrast, ions identified with the Low CE profile often resulted in low-scoring or overfragmented spectra in measurements done with higher collision energy settings. These results highlight the benefit of characterizing samples with distinct collision energy profiles to expand the coverage of identifiable peptides in a sample. Although exposure of precursor ions to distinct collision energy profiles was required to achieve ideal fragmentation, by design this approach does not produce good XSMs for all XLs in all replicates. Here is where careful matching between runs is facilitated by the consistent detection of precursor ion data and their retention time, indexed against iRT standards. Looking forward, implementing further DDA gas phase fractionation^27^ or off-line fractionation^28^ could be used to enhance spectral library comprehensiveness with the workflow proposed here.

Moreover, by using the iRTs of XLs it is possible to capitalize on the typically low signal yields of cross-linking reactions, utilizing them as indicators of authentic XL presence. It involves contrasting the iRT of XLs in negative controls where no cross-linker has been added to ensure the XL ID is not assigned to other matrix signals. This method is especially valuable for discerning unique, low-intensity species linked to XSMs that were marginally accepted by identification software. Once confirmed as unique to the cross-linking reaction, these ambiguous, low-intensity ions can be prioritized for reanalysis using targeted methods. While not addressed in this study, this approach could also be adapted for entirely untargeted analyses to detect unique cross-linking chromatographic features, with ID assignment as a subsequent step.

For example, in a current cross-linking study between neuropeptide Y (NPY) and its receptor Y_2_R, the peptide-centric method provided a reliable means to detect XL sites at the N-terminus of Y_2_R (manuscript in preparation). These sites belong to a region predicted to be intrinsically disordered, showcasing the ability of this approach to reveal interactions that have been intractable to other structural proteomics techniques.

Despite its limitations, DDA remains widely used in XL-MS because it offers clearer determination of fragment ion origins, simplifying the interpretation of complex XL fragmentation patterns. In this technical note and accompanying tutorial materials (Supporting Information), we demonstrate how to exploit some of these limitations by matching identifications between LC-MS/MS runs, a common practice in other proteomics fields.^29^ However, we present this approach in a platform that facilitates critical visual analysis of mass spectrometry data to prevent false transfer of IDs and promote a transition toward peptide-centric evaluation of XL-MS data.

### Framework for DIA-XL-MS Data Analysis in Skyline and Integrative Data Processing

Recent workflows have shown the processing of XL-MS results acquired with DIA using DDA spectral libraries for a peptide-centric data analysis.^9,10^ The presented workflow leverages Skyline’s visualization tools for DIA data interrogation. As a proof of concept, a Skyline document examined DIA-PASEF data from a separately prepared BSA-DSBU digest, measured weeks apart. This data set can be found in https://panoramaweb.org/XL-MS_MeroX_Skyline.url. The use of validated iRTs from DDA measurements was key to aligning results, accommodating retention time shifts from changes to the LC system, i.e., change of trap and analytical column. iRTs’ role in data set alignment tests uniformity across different sample preparations, addressing challenges for reliable quantitative XL-MS studies.^1^

DIA data holds promise for improving reproducibility and selectivity in peak detection, especially with peptide-specific fragment ions. However, identifying low-intensity XLs solely focusing on fragment ions presents a challenge. DDA remains essential for generating spectral libraries to analyze DIA data, facilitated by Skyline’s vendor-neutral platform and compatibility with various data sources once formatted to ProXL. This integrated approach, validated as an effective spectrum-centric filter for XLs in studies like Matzinger et al.,^21^ enhances the robustness and authenticity of identified XLs. The comprehensive workflow, utilizing accessible tools such as DataAnalysis, MeroX, ProXL, and Skyline, signifies a paradigm shift in the cross-linking community’s data analysis methods. It not only demonstrates the collaborative potential of instrument vendors and open-source platforms but also establishes a replicable and reliable path for unraveling complex proteome interactions, advancing toward more nuanced proteomic exploration.

## Conclusion

Advancements in XL-MS, driven by peptide-centric validation and interrogation of quantitative queries, signify progress in analyzing protein interactions. The integration of PASEF for DDA and DIA pipelines sets a new standard for identifying and quantifying XLs. Despite the challenges in DIA-PASEF data analysis for XLs, this technical note outlines effective strategies for overcoming these obstacles. The creation of comprehensive DDA spectral libraries from multiple search engines in Skyline and empirical retention time indexing are crucial in this dynamic field, ensuring rigorous and innovative analysis.

## Data Availability

Raw data are publicly available with ProteomeXchange identifier PXD047307.

## Supporting Information Available

Tutorial: Cross-linking mass spectrometry with timsTOF DDA-PASEF using MeroX and Skyline (PDF)
